# Assessment of the Appropriateness of Antibiotic Prescriptions for Infection Prophylaxis Before Dental Procedures, 2011 to 2015

**DOI:** 10.1001/jamanetworkopen.2019.3909

**Published:** 2019-05-31

**Authors:** Katie J. Suda, Gregory S. Calip, Jifang Zhou, Susan Rowan, Alan E. Gross, Ronald C. Hershow, Rose I. Perez, Jessina C. McGregor, Charlesnika T. Evans

**Affiliations:** 1 College of Pharmacy, University of Illinois at Chicago; 2 Center of Innovation for Complex Chronic Healthcare, Edward Hines, Jr VA Hospital, Hines, Illinois; 3 College of Dentistry, University of Illinois at Chicago; 4 School of Public Health, University of Illinois at Chicago; 5 College of Medicine, University of Illinois at Chicago; 6 Oregon State University, Corvallis; 7 College of Pharmacy, Oregon Health and Science University, Portland; 8 Northwestern University Feinberg School of Medicine, Chicago, Illinois

## Abstract

**Importance:**

Antibiotics are recommended before certain dental procedures in patients with select comorbidities to prevent serious distant site infections.

**Objective:**

To assess the appropriateness of antibiotic prophylaxis before dental procedures using Truven, a national integrated health claims database.

**Design, Setting, and Participants:**

Retrospective cohort study. Dental visits from 2011 to 2015 were linked to medical and prescription claims from 2009 to 2015. The dates of analysis were August 2018 to January 2019. Participants were US patients with commercial dental insurance without a hospitalization or extraoral infection 14 days before antibiotic prophylaxis (defined as a prescription with ≤2 days’ supply dispensed within 7 days before a dental visit).

**Exposures:**

Presence or absence of cardiac diagnoses and dental procedures that manipulated the gingiva or tooth periapex.

**Main Outcomes and Measures:**

Appropriate antibiotic prophylaxis was defined as a prescription dispensed before a dental visit with a procedure that manipulated the gingiva or tooth periapex in patients with an appropriate cardiac diagnosis. To assess associations between patient or dental visit characteristics and appropriate antibiotic prophylaxis, multivariable logistic regression was used. A priori hypothesis tests were performed with an α level of .05.

**Results:**

From 2011 to 2015, antibiotic prophylaxis was prescribed for 168 420 dental visits for 91 438 patients (median age, 63 years; interquartile range, 55-72 years; 57.2% female). Overall, these 168 420 dental visits were associated with 287 029 dental procedure codes (range, 1-14 per visit). Most dental visits were classified as diagnostic (70.2%) and/or preventive (58.8%). In 90.7% of dental visits, a procedure was performed that would necessitate antibiotic prophylaxis in high-risk cardiac patients. Prevalent comorbidities include prosthetic joint devices (42.5%) and cardiac conditions at the highest risk of adverse outcome from infective endocarditis (20.9%). Per guidelines, 80.9% of antibiotic prophylaxis prescriptions before dental visits were unnecessary. Clindamycin was more likely to be unnecessary relative to amoxicillin (odds ratio [OR], 1.10; 95% CI, 1.05-1.15). Prosthetic joint devices (OR, 2.31; 95% CI, 2.22-2.41), tooth implant procedures (OR, 1.66; 95% CI, 1.45-1.89), female sex (OR, 1.21; 95% CI, 1.17-1.25), and visits occurring in the western United States (OR, 1.15; 95% CI, 1.06-1.25) were associated with unnecessary antibiotic prophylaxis.

**Conclusion and Relevance:**

More than 80% of antibiotics prescribed for infection prophylaxis before dental visits were unnecessary. Implementation of antimicrobial stewardship in dental practices is an opportunity to improve antibiotic prescribing for infection prophylaxis.

## Key Points

**Question:**

Are antibiotics appropriately prescribed for infection prophylaxis before dental procedures?

**Findings:**

In this cohort study of 91 438 patients who received antibiotic prophylaxis for 168 420 dental visits from 2011 to 2015, a total of 90.7% of dental visits had manipulation of the gingiva or tooth periapex, but only 20.9% of patients had a cardiac condition at the highest risk of adverse outcome from infective endocarditis. Therefore, 80.9% of antibiotic prophylaxis prescriptions were discordant with guidelines.

**Meaning:**

Most antibiotics prescribed for infection prophylaxis before dental procedures are unnecessary.

## Teaser

This cohort study assesses the appropriateness of antibiotic prophylaxis prescriptions for infection prophylaxis before dental procedures using Truven, a US national integrated health claims database.

## Introduction

Dentists prescribe 1 in 10 antibiotic prescriptions and are the top specialty prescriber of antibiotics in the United States.^1^ Antibiotic prescribing by dentists is common, comprising almost 60% of prescriptions to Medicare Part D beneficiaries.^2^ While decreases in prescribing of antibiotics have been observed nationally, dental prescribing has remained steady.^3^ This is despite changes in clinical guidelines narrowing the indications for antibiotic prophylaxis before dental procedures.^4,5,6,7,8^

Prior infection prophylaxis guidelines recommended that patients with certain conditions (ie, patients with recent prosthetic joint implants) receive antibiotic prophylaxis before a dentist visit. The rationale for prophylaxis was that patients with these conditions have an increased risk for serious distant site infections (eg, infective endocarditis and prosthetic joint infections) secondary to bacteremia introduced during dental care. However, guidelines for the use of antibiotics for the prevention of infective endocarditis and prosthetic joint infections were revised in 2007 and 2013, respectively.^5,8^ The rationale for these revisions was secondary to poor evidence on the effectiveness of antibiotic prophylaxis, lack of an association between endocarditis and joint infections and dental care, and the risk of antibiotic-associated adverse events.^5,6,9^ Antibiotic resistance, risk of *Clostridioides difficile* infection, and general adverse effects outweigh any potential benefit, which is likely to be small.^6,9,10^ Therefore, antibiotics before dental procedures are only recommended per guidelines in patients with cardiac conditions at the highest risk of adverse outcome from infective endocarditis undergoing invasive dental procedures.^5^

While studies in outpatient primary medical care settings have demonstrated that 30% of antibiotics prescribed are unnecessary,^11^ no study has evaluated the appropriateness of antibiotic prescribing by dentists. Therefore, the objective of this study was to assess the appropriateness of antibiotic prophylaxis before dental procedures.

## Methods

In this retrospective cohort study, we performed an analysis of adult patients with visits to a dentist from 2011 to 2015 using Truven MarketScan commercial claims and encounters, Medicare supplemental, and coordination of benefits. Truven is a national integrated health claims database of deidentified outpatient medical, hospital, prescription, and dental claims. While the medical claims are nationally representative of the insured US population in terms of age, sex, and geographic area,^12,13,14,15^ the dental domain is a convenience sample of 8 million persons with enrollment in both medical (commercial insurance or Medicare) and dental (commercial) health plans. This is the only national data set with detailed data on dental claims and links medical and prescription claims.^16^ Per other health services research performed by the American Dental Association,^16,17,18,19,20^ we collected individual-level demographics (patient age and sex), inpatient and outpatient medical diagnoses (in *International Classification of Diseases*, *Ninth Revision [ICD-9]* or *International Statistical Classification of Diseases*, *10th Revision*, *Clinical Modification [ICD-10-CM]* format), medical procedures (in *Current Procedural Terminology* and Healthcare Common Procedure Coding System format), retail and mail-order prescription claims, and dental claims (in *Code on Dental Procedures and Nomenclature [CDT]* format). Dental visit *CDT* codes were aggregated into categories per a standardized coding structure established by the American Dental Association. Because multiple *CDT* codes could be coded for a visit, our analyses assessed visits with a specific *CDT* category coded as compared with visits without the *CDT* category coded. For all analyses herein, *ICD-9* codes before October 1, 2015, were converted to *ICD-10-CM* codes per guidance from the Centers for Disease Control and Prevention.^21^ This study followed the Strengthening the Reporting of Observational Studies in Epidemiology (STROBE) reporting guideline. The University of Illinois at Chicago Investigational Review Board deemed that this study was exempt from review and informed consent.

### Study Population

Prescription claims were identified in the Truven prescription claims data, and dental procedures were identified in the Truven dental commercial claims data (2011-2015), with prevalence of cardiac or other conditions identified in the Truven medical and hospital claims data (2009-2015) before the dispensing of antibiotic prescriptions for the dental visit. The dates of analysis were August 2018 to January 2019. Eligible study patients were those with 12 months of continuous enrollment in plans with medical and prescription coverage and 30 days with dental coverage before and including the date of the visit with a dentist. We identified patients with a prescription claim for any systemic antibiotic with a days’ supply of 2 days or less that occurred within 7 days before the dental visit. In this study, these antibiotics were defined as being prescribed for preprocedural infection prophylaxis and meet the standard from prior studies, manual review of dental records, and per guidance from clinical experts.^22^ Antibiotics with a days’ supply of 2 days or less dispensed within 7 days before the dental visit met our definition of use for antibiotic prophylaxis vs treatment of an oral or systemic infection (requiring ≥3 days’ treatment).^22^ We further excluded visits at which patients had a recent hospitalization or active or recent extraoral infection (eg, recent respiratory tract infection) defined by diagnosis codes from any outpatient encounter. A recent hospitalization or extraoral infection was defined as occurring within 14 days before the antibiotic dispense date.^23,24^

**Figure 1.**
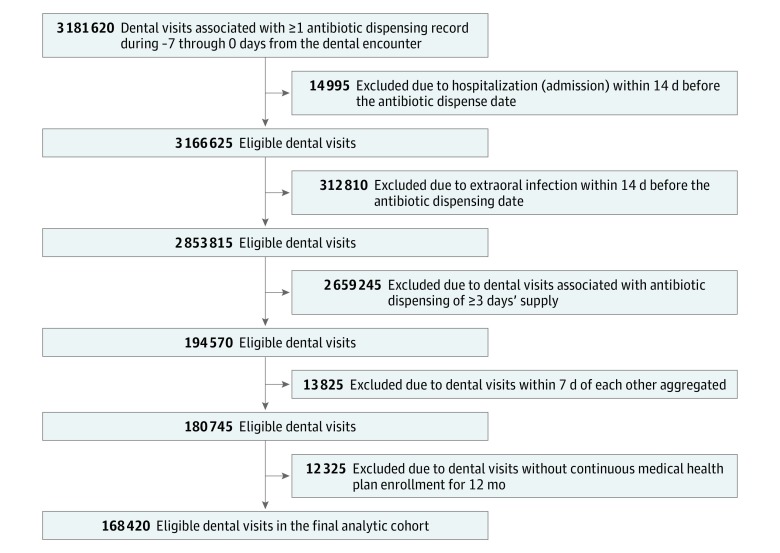
Study Flowchart Derivation of the study population is shown.

Because dental visits are typically connected (eg, a tooth requiring extraction is identified at one visit but is extracted at a second visit), we combined all dental visits occurring within 7 days from each other into a single observation or an episode of care. All codes from each visit were combined and represented in the episode of care. For example, a diagnostic dental visit that is followed by a tooth implant dental visit 3 days later is combined into an episode of care with diagnostic and implant codes. Clustering visits into episodes of care excluded 13 825 visits (6.8% of the cohort had 2 visits, and 0.3% had ≥3 visits) (Figure 1). Of the visits clustered into episodes of care, 23.8% had a visit the next day (1 day apart), 14.4% were 2 days apart, 9.6% were 3 days apart, 8.9% were 4 days apart, 11.2% were 5 days apart, 13.5% were 6 days apart, and 18.5% were 7 days apart. Broadening the episode of care definition to 14 and 30 days linked visits that were unlikely to be relevant clinically and thus captured few additional visits. Episodes of care are reported as visits herein but are in fact visits collapsed into episodes of care as described previously.

### Study Definitions

Guidelines on the use of antibiotics for infective endocarditis prophylaxis are based on specific planned dental procedures and medical history. Recommendations in current and previous guidelines for the use of antibiotic prophylaxis before dental procedures are summarized in Table 1.^5,8,25^ For antibiotic prophylaxis to be indicated for the prevention of infective endocarditis, both the dental procedure and the medical history need to be consistent with guidelines.^26^ Dental procedures for which infective endocarditis prophylaxis is considered appropriate are all procedures that involve manipulation of gingival tissue or the periapical region of teeth or perforation of the oral mucosa (referred to as gingival manipulation herein).^5^ All *CDT*, *Current Procedural Terminology*, and Healthcare Common Procedure Coding System codes associated with dental visits during the study period were assessed for gingival manipulation by 6 general and 6 specialty dentist raters. When raters disagreed, consensus was determined by an additional rater team of 3 general dentists and 1 oral surgeon. Patients with cardiac conditions that are associated with the highest risk of adverse outcome from endocarditis are recommended to receive antibiotic prophylaxis for dental procedures that involve gingival manipulation. Cardiac conditions for which antibiotics are indicated per guidelines include prosthetic cardiac valve or material used for cardiac valve repair, previous infective endocarditis, certain congenital heart diseases, and cardiac transplant recipients with cardiac valvulopathy.^5^ Cardiac conditions occurring from 2009 until the date the antibiotic was dispensed were evaluated and defined consistent with previous investigators.^8,27,28,29,30,31,32,33,34,35,36,37,38,39^

**Table 1.** Guideline Summary on the Use of Antibiotic Prophylaxis Before Dental Procedures

Variable	Year Published	Criteria for Antibiotic Prophylaxis	Recommendation
Current guidelines in patients with cardiac conditions at the highest risk for infective endocarditis published by the American Heart Association^5^	2007	Prosthetic cardiac valve or material used for valve repair	One dose of an antibiotic is recommended before dental visits with procedures that involve manipulation of gingival tissue or the periapical region of teeth or perforation of the oral mucosa
		Previous infective endocarditis	
		Certain congenital heart diseases^a^	
		Cardiac transplants with cardiac valvulopathy^a^	
Current guidelines in patients with prosthetic joints published by the American Academy of Orthopaedic Surgeons and the American Dental Association^8^	2013 (Released online in 2012)	None	Antibiotic prophylaxis is not recommended in patients with prosthetic joints
Prior guidelines in patients with prosthetic joints published by the American Dental Association and the American Academy of Orthopaedic Surgeons^25^	2003	All patients during the first 2 y after joint placement	One dose of an antibiotic is recommended before dental visits with procedures that have a higher bacteremia risk
		Immunocompromised conditions (inflammatory arthropathies and drug-induced or radiotherapy-induced immunosuppression)	
		History of prosthetic joint infections	
		Malnourishment	
		Hemophilia	
		HIV or AIDS	
		Type 1 diabetes^a^	
		Cancer	

^a^ Because of the difficulty in identifying these conditions in claims data, all patients with congenital heart disease, cardiac transplantation (not limited to those with cardiac valvulopathy), and type 1 and type 2 diabetes were included in these categories in our analyses.

Antibiotics are not recommended for prosthetic joint infection prophylaxis,^6,7,8^ defined as recent (prior 2 years) or historical claims (Table 1). Before 2012, guidelines for prosthetic joint infection prophylaxis recommended antibiotic prophylaxis in patients with select comorbidities (eg, prosthetic joint plus cancer) (Table 1). Hospital and medical claims of any prosthetic joint implant or complication (eg, revision or infection) occurring before the antibiotic dispense date were defined consistent with previous investigators.^40,41,42,43^ Therefore, “appropriate” antibiotic prophylaxis was defined as a prescription dispensed before a dental visit with a procedure that manipulated the gingiva or tooth periapex (as defined by dental raters described in the previous paragraph) in patients with an appropriate cardiac diagnosis as indicated by guidelines.^5^ In the absence of cardiac conditions, antibiotic prophylaxis was considered unnecessary in the primary analysis.^6,7,8^

Given the varying guidelines and recommendations occurring over the course of the study period (updated prosthetic joint infection prophylaxis guidelines were published in 2013 and reaffirmed in 2015),^7,8^ we conducted sensitivity analyses varying the definition of what would qualify as an indication for joint-related prophylaxis (1) defining a 2-year window of appropriate antibiotic prescription after prosthetic joint placement (the highest-risk period for infection)^7^ and (2) using prior guidelines^25^ per recommendations by Lockhart.^44^ Other sensitivity analyses estimating the appropriateness of antibiotic prophylaxis included varying selection criteria within plausible scenarios of (1) continuous medical plan enrollment throughout the entire study period, (2) stratified years of estimates to reflect changes in practice and guideline recommendations, and (3) stratification of patients with and without a history of prosthetic joint diagnoses or procedures.

### Statistical Analysis

Descriptive characteristics of groups with visits identified as having an appropriate use of antibiotic prophylaxis and those that did not were compared using independent-samples *t* test for continuous variables and χ^2^ test for categorical variables. Missing data (55 observations) were included in the analysis and are labeled in the multivariable analysis as unknown. Multivariable logistic regression models were used to calculate adjusted odds ratios (ORs) and 95% CIs with robust standard errors for association between patient-level and visit-level characteristics and appropriate antibiotic use. Also, in analyses that included multiple visits per patient (54.3% in the primary analysis), generalized estimating equations with a first-order autoregressive working matrix were used to estimate corresponding ORs and 95% CIs.^45^ Variables significant in unadjusted analyses or identified as factors associated with unnecessary antibiotic prophylaxis were included in the model. The most parsimonious model was selected by only including those final variables significantly associated with prescribing. SAS, version 9.4 (SAS Institute Inc), was used for all analyses. A priori hypothesis tests were performed with a 2-sided α level of .05.

## Results

More than 3 million dental visits and antibiotic prescriptions were identified in the Truven commercial dental database during the study period of 2011 to 2015. After applying inclusion and exclusion criteria, 168 420 eligible dental visits (or episodes of care) with antibiotic prophylaxis for 91 438 unique patients were included in this analysis (Figure 1). The characteristics of the sample are listed in Table 2. The median age of the cohort was 63 years (interquartile range, 55-72 years) and was majority female (57.2%). Overall, these 168 420 dental visits were associated with 287 029 dental procedure codes (range, 1-14 per visit). Most visits with antibiotic prophylaxis occurred in the midwestern United States (46.9%), followed by the southern United States (31.5%), and occurred in an urban setting (56.7%). Most dental procedures conducted during the dental visits with antibiotic prophylaxis were classified as diagnostic (70.2%), followed by preventive (58.8%), and involved some type of gingival manipulation and/or mucosal incision (90.7%). Comorbidities were common in the cohort, especially prosthetic joint devices (42.5%) and cardiac conditions at the highest risk of adverse outcome from infective endocarditis (20.9%). Health care use in the previous 6 months was common, with half having a primary care visit (50.7%) and most having a specialty care visit (80.4%).

**Table 2.** Descriptive Characteristics of Eligible Dental Visits (2011-2015) and Unadjusted Associations With the Appropriateness of Antibiotic Prophylaxis Among 168 420 Dental Visits

Variable	Total (N = 168 420)	Appropriate Antibiotic Prophylaxis (n = 32 243)	Unnecessary Antibiotic Prophylaxis (n = 136 177)
Age at visit, y			
Mean (SD)	62.2 (14.8)	63.3 (15.9)	61.9 (14.5)
Median (IQR)	63 (55-72)	64 (55-75)	62 (55-71)
Age category at visit, y, No. (%)			
18-34	9175 (5.4)	1941 (6.0)	7234 (5.3)
35-44	8276 (4.9)	1782 (5.5)	6494 (4.8)
45-54	22 749 (13.5)	4129 (12.8)	18 620 (13.7)
55-64	57 979 (34.4)	9217 (28.6)	48 762 (35.8)
≥65	70 241 (41.7)	15 174 (47.1)	55 067 (40.4)
Female sex, No. (%)	96 262 (57.2)	17 081 (53.0)	79 181 (58.1)
US region, No. (%)^a^			
Northeast	22 695 (13.5)	4947 (15.3)	17 748 (13.0)
Midwest	78 948 (46.9)	14 374 (44.6)	64 574 (47.4)
South	52 987 (31.5)	10 781 (33.4)	42 206 (31.0)
West	13 735 (8.2)	2134 (6.6)	11 601 (8.5)
Rural vs urban, No. (%)			
Rural	72 905 (43.3)	15 399 (47.8)	57 506 (42.2)
Urban	95 515 (56.7)	16 844 (52.2)	78 671 (57.8)
Antibiotic prescribed, No. (%)^b^			
Amoxicillin	116 908 (69.4)	24 466 (75.9)	92 442 (67.9)
Clindamycin	27 031 (16.0)	5066 (15.7)	21 965 (16.1)
Cephalexin	13 879 (8.2)	1470 (4.6)	12 409 (9.1)
Azithromycin	5297 (3.1)	694 (2.2)	4603 (3.4)
Penicillin	3620 (2.1)	505 (1.6)	3115 (2.3)
Doxycycline	1656 (1.0)	135 (0.4)	1521 (1.1)
Other^c^	4666 (2.8)	624 (1.9)	4042 (3.0)
ADA dental procedure category, No. (%)^d^			
Diagnostic	118 215 (70.2)	24 899 (77.2)	93 316 (68.5)
Preventive	99 059 (58.8)	21 902 (67.9)	77 157 (56.7)
Restorative	30 955 (18.4)	4884 (15.1)	26 071 (19.1)
Oral and maxillofacial surgery	10 808 (6.4)	1611 (5.0)	9197 (6.8)
Periodontics	11 995 (7.1)	2431 (7.5)	9564 (7.0)
Adjunctive general services	4842 (2.9)	607 (1.9)	4235 (3.1)
Endodontics	3503 (2.1)	706 (2.2)	2797 (2.1)
Implant services	2456 (1.5)	168 (0.5)	2288 (1.7)
Prosthodontics	2147 (1.3)	285 (0.9)	1862 (1.4)
Orthodontics	224 (0.1)	10 (0.0)	214 (0.2)
Maxillofacial prosthetics	22 (0.0)	1 (0.0)	21 (0.0)
Category not available	2803 (1.7)	7 (0.0)	2796 (2.1)
Gingival manipulation, No. (%)	152 711 (90.7)	32 243 (100.0)	120 468 (88.5)
Previsit conditions, No. (%)			
Prosthetic joint device	71 651 (42.5)	8041 (24.9)	63 610 (46.7)
Cardiac condition^e^	35 224 (20.9)	32 243 (100.0)	2981 (2.2)
Diabetes^f^	38 421 (22.8)	7986 (24.8)	30 435 (22.3)
Immunocompromised state^g^	9211 (5.5)	1733 (5.4)	7478 (5.5)
Preindex health service use^h^			
PCP visits, mean (SD)	1.7 (3.5)	1.9 (4.0)	1.6 (3.4)
Any PCP visit, No. (%)	85 399 (50.7)	16 504 (51.2)	68 895 (50.6)
Specialist visits, mean (SD)	7.3 (9.9)	8.4 (10.6)	7.0 (9.8)
Any specialist visit, No. (%)	135 375 (80.4)	27 144 (84.2)	108 231 (79.5)
ED visits, mean (SD)	0.2 (0.8)	0.3 (0.9)	0.2 (0.7)
Any ED visit, No. (%)	24 361 (14.5)	6199 (19.2)	18 162 (13.3)
Admissions, mean (SD)	0.2 (0.4)	0.2 (0.5)	0.1 (0.4)
Any admission, No. (%)	21 985 (13.1)	4530 (14.0)	17 455 (12.8)

Abbreviations: ADA, American Dental Association; ED, emergency department; IQR, interquartile range; PCP, primary care provider.

^a^ A total of 55 observations were missing, 7 in the appropriate group and 48 in the unnecessary group.

^b^ There could be multiple antibiotic dispensing records associated with the same visit (2.7% had >1 antibiotic associated with the dental visit).

^c^ Other antibiotics include the following: ampicillin (n = 358), cefaclor (n = 30), cefadroxil (n = 235), cefazolin (n = 2), cefdinir (n = 37), cefixime (n = 49), cefoxitin (n = 5), cefpodoxime (n = 20), cefprozil (n = 9), ceftazidime (n = 3), ceftriaxone (n = 54), cefuroxime (n = 86), ciprofloxacin (n = 1336), clarithromycin (n = 336), demeclocycline (n = 2), dicloxacillin (n = 15), erythromycin (n = 860), gemifloxacin (n = 3), levofloxacin (n = 439), lincomycin (n = 1), linezolid (n = 7), minocycline (n = 102), moxifloxacin (n = 117), ofloxacin (n = 51), sulfamethoxazole-trimethoprim (n = 434), tetracycline (n = 19), trimethoprim (n = 38), and vancomycin (n = 19).

^d^ The ADA has a standardized system to group *Code on Dental Procedures and Nomenclature (CDT)* codes (dental procedure codes) into categories (shown in the Table). There could be multiple procedures performed during the same visit. The ADA does not include *Current Procedural Terminology (CPT)* codes and Healthcare Common Procedure Coding System (HCPCS) codes in their standard ADA dental procedure categories. The *CPT* and HCPCS codes are included in “category not available.”

^e^ Cardiac conditions were defined according to the study by Wilson et al^5^ as those at the highest risk of infective endocarditis.

^f^ The diabetes category includes those with type 1 and type 2 diabetes.

^g^ Immunocompromised state was defined according to previous guidelines from the ADA and the American Academy of Orthopaedic Surgeons.^25^

^h^ Health service use assessed over the 6-month predental visit period, not accounting for enrollment in dental or medical plans. We defined outpatient clinic visits with a health care provider type of nurse practitioners, physician assistants, or medical doctors. Medical doctors with a specialty of internal medicine or family medicine were included as PCPs. Other types of clinical encounters were defined as a specialist visit and may include health care encounters without a medical care provider (eg, nurse visit or laboratory visit).

### Unadjusted Analysis

**Figure 2.**
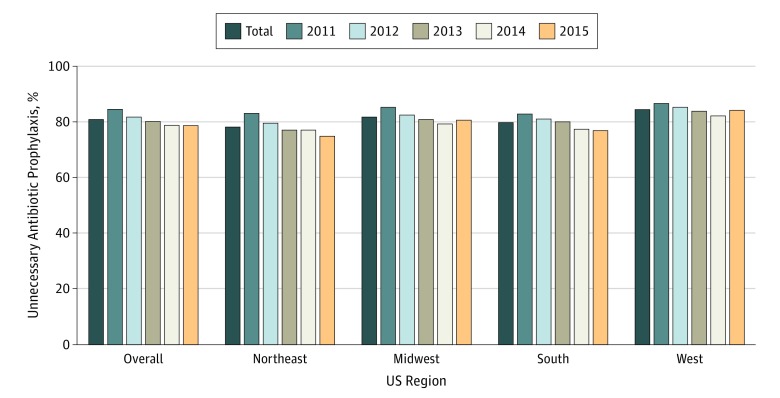
Geographic Variation in Unnecessary Antibiotic Prophylaxis, 2011-2015 Results from the unadjusted analysis are shown.

Of the 168 420 eligible dental visits with antibiotic prophylaxis, the most frequent antibiotics prescribed were amoxicillin (69.4%), followed by clindamycin (16.0%) (Table 2). Only 19.1% of antibiotics prescribed were appropriate; therefore, 80.9% of antibiotic prophylaxis prescriptions before dental visits were discordant with guidelines. Those with unnecessary antibiotic prophylaxis had a lower percentage of amoxicillin prescribed but a higher percentage of cephalexin and other types of antibiotics than those with appropriate antibiotic prophylaxis. Compared with those visits with appropriate antibiotic prophylaxis, visits with unnecessary antibiotic prophylaxis were associated with a higher percentage of women, urban locations, and prosthetic joints but a lower percentage of diagnostic and preventive dental procedure categories, gingival manipulation, cardiac conditions, and previous health care use. There was also significant variation by US Census geographic region and the appropriateness of antibiotic prophylaxis. The highest unnecessary prescribing was observed in the West (84.5% of antibiotic prophylaxis prescriptions were unnecessary in the West) and the lowest in the Northeast (78.2% of antibiotic prophylaxis prescriptions were unnecessary in the Northeast). Unnecessary antibiotic prophylaxis decreased over time between 2011 and 2015 from 84.5% to 78.8% overall and across all geographic regions (*P* < .001) (Figure 2).

### Adjusted Analysis

In multivariable analyses, unnecessary antibiotic prophylaxis decreased over time (Table 3). Patients 65 years and older and those aged 35 to 44 years had lower odds of being prescribed unnecessary antibiotic prophylaxis compared with patients aged 18 to 34 years, while women (vs men; odds ratio [OR], 1.21; 95% CI, 1.17-1.25) had higher odds of unnecessary prescribing. Significant regional variation remained: compared with the Midwest, the Northeast and South had lower odds of unnecessary antibiotic prophylaxis, while the West (OR, 1.15, 95% CI, 1.06-1.25) had higher odds of unnecessary antibiotic prophylaxis. In addition, being in a rural location was associated with lower odds of unnecessary antibiotic prophylaxis. Dental procedures defined as diagnostic, preventive, oral and maxillofacial surgery, periodontics, and endodontics had lower odds of unnecessary antibiotic prophylaxis compared with those without these *CDT* procedure categories; adjunctive general services, implant services (OR, 1.66; 95% CI, 1.45-1.89), prosthodontics, orthodontics, and maxillofacial prosthetics had higher odds of unnecessary prescribing. Unnecessary antibiotic prophylaxis was less common in patients with diabetes, immunocompromised conditions, or health care use in the previous 6 months, while having a prosthetic joint device (OR, 2.31; 95% CI, 2.22-2.41) was associated with unnecessary prescribing compared with those not having a prosthetic joint. Finally, unnecessary antibiotic prophylaxis was most associated with antibiotics falling into the “other” category, followed by clindamycin (OR 1.10; 95% CI, 1.05-1.15), compared with amoxicillin.

**Table 3.** Multivariable Analysis of Factors Associated With Unnecessary Antibiotic Prophylaxis Among 168 420 Dental Visits

Variable	Odds Ratio (95% CI)
Age category at visit, y	
18-34	1 [Reference]
35-44	0.90 (0.81-0.99)
45-54	0.94 (0.86-1.03)
55-64	0.93 (0.85-1.01)
≥65	0.71 (0.65-0.78)
Sex	
Male	1 [Reference]
Female	1.21 (1.17-1.25)
US region	
Northeast	0.76 (0.72-0.81)
Midwest	1 [Reference]
South	0.88 (0.85-0.92)
West	1.15 (1.06-1.25)
Unknown	1.81 (0.64-5.06)
Rural vs urban	
Rural	0.78 (0.75-0.82)
Urban	1 [Reference]
Year of visit	
2011	1 [Reference]
2012	0.81 (0.79-0.84)
2013	0.70 (0.67-0.72)
2014	0.59 (0.56-0.61)
2015	0.53 (0.51-0.55)
Antibiotic prescribed	
Amoxicillin	1 [Reference]
Clindamycin	1.10 (1.05-1.15)
Other^a^	1.70 (1.61-1.79)
ADA dental procedure category^b^	
Diagnostic	0.90 (0.87-0.92)
Preventive	0.62 (0.60-0.65)
Restorative	0.98 (0.95-1.02)
Oral and maxillofacial surgery	0.72 (0.68-0.75)
Periodontics	0.65 (0.62-0.68)
Adjunctive general services	1.47 (1.32-1.62)
Endodontics	0.62 (0.58-0.65)
Implant services	1.66 (1.45-1.89)
Prosthodontics	1.65 (1.41-1.95)
Orthodontics	3.04 (1.06-8.68)
Maxillofacial prosthetics	1.53 (1.04-2.27)
Previsit conditions	
Prosthetic joint device	2.31 (2.22-2.41)
Diabetes	0.87 (0.83-0.91)
Immunocompromised state	0.91 (0.85-0.98)
Preindex health service use (yes or no)^c^	
PCP visits	0.96 (0.94-0.98)
Specialist visits	0.86 (0.84-0.88)
ED visits	0.91 (0.88-0.93)
Admissions	0.86 (0.83-0.88)

Abbreviations: ADA, American Dental Association; ED, emergency department; PCP, primary care provider.

^a^ Other antibiotics include the following: ampicillin (n = 358), cefaclor (n = 30), cefadroxil (n = 235), cefazolin (n = 2), cefdinir (n = 37), cefixime (n = 49), cefoxitin (n = 5), cefpodoxime (n = 20), cefprozil (n = 9), ceftazidime (n = 3), ceftriaxone (n = 54), cefuroxime (n = 86), ciprofloxacin (n = 1336), clarithromycin (n = 336), demeclocycline (n = 2), dicloxacillin (n = 15), erythromycin (n = 860), gemifloxacin (n = 3), levofloxacin (n = 439), lincomycin (n = 1), linezolid (n = 7), minocycline (n = 102), moxifloxacin (n = 117), ofloxacin (n = 51), sulfamethoxazole-trimethoprim (n = 434), tetracycline (n = 19), trimethoprim (n = 38), and vancomycin (n = 19).

^b^ The ADA has a standardized system to group dental procedures codes (*Code on Dental Procedures and Nomenclature* codes) into categories (shown in the Table). There could be multiple procedures performed during the same visit. The ADA does not include *Current Procedural Terminology* codes and Healthcare Common Procedure Coding System codes in their standard ADA dental procedure categories.

^c^ Health service use assessed over the 6-month predental visit period, not accounting for enrollment in dental or medical plans. We defined outpatient clinic visits with a health care provider type of nurse practitioners, physician assistants, or medical doctors. Medical doctors with a specialty of internal medicine or family medicine were included as PCPs. Other types of clinical encounters were defined as a specialist visit and may include health care encounters without a medical care provider (eg, nurse visit or laboratory visit).

Sensitivity analyses were conducted to assess whether our findings were robust to assumptions of selection criteria within a plausible range. In the event that medical coding missed a cardiac condition diagnosis, we limited our cohort to those patients continuously enrolled in the medical plan from 2009 until the date of the visit. In this continuous enrollment analysis, 60.1% (n = 101 231) of the cohort were included, and 80.2% of antibiotic prophylaxis prescriptions were defined as unnecessary. The 2003 guidelines for the prevention of prosthetic joint infections also changed during our study period (updated guidelines were published in 2013 [released online in 2012]) (Table 1).^8,25^ The results were similar after modifying our definition of appropriate antibiotic prophylaxis with recommendations in the 2003 prosthetic joint infection guidelines^25^ (75.7% of antibiotic prophylaxis prescriptions were unnecessary overall [76.7% in 2011-2013 and 74.1% in 2014-2015]). The highest-risk period of a prosthetic joint infection after joint implant placement is 2 years.^25^ After including prosthetic joint placement within 2 years in our definition of appropriate antibiotic prophylaxis, 80.9% of antibiotics prescribed for infection prophylaxis before dental visits were unnecessary. A subanalysis of the cohort with visits clustered into episodes of care (n = 13 825), 82.7% of patients at the first visit, 84.6% at the second visit, and 84.3% at the third visit received unnecessary antibiotic prophylaxis. Stratifying the overall cohort by the presence or absence of a prosthetic joint, 11.2% of patients with a prosthetic joint received appropriate antibiotic prophylaxis secondary to a concomitant cardiac condition (n = 71 651). After excluding those with a prosthetic joint, the percentage of visits with unnecessary antibiotic prophylaxis decreased to 75.0% (n = 96 769). Similar characteristics were found to be associated with unnecessary antibiotic prophylaxis as identified in the full analysis (eTables 1, 2, and 3 in the Supplement).

## Discussion

Our results demonstrate that most antibiotics prescribed for infection prophylaxis before dental visits are unnecessary. These findings are concerning because dentists prescribe a significant proportion of antibiotics and are the top prescribers of clindamycin in the United States.^1,46^ Antibiotics prescribed for infection prophylaxis by dentists have been associated with community-associated *C difficile* infection.^27,47,48^ One dose of clindamycin has an equivalent risk of *C difficile* compared with a prolonged course.^49^ Therefore, it is alarming that clindamycin was more likely to be inappropriately prescribed than amoxicillin. However, there was a significant decrease in antibiotic prophylaxis over the study period. This may indicate that the 2013 guidelines for the prevention of prosthetic joint infections are being applied to patient care.

These results are consistent with those from other countries, where 58% to 81% of dental antibiotic prescriptions are inconsistent with guidelines, particularly for infection prophylaxis.^50,51,52,53,54,55,56,57,58^ However, dentists are knowledgeable about and generally satisfied with the antibiotic prophylaxis guidelines.^59^ Regardless, 70% of dentists surveyed reported prescribing antibiotic prophylaxis when not indicated.^59^ Dentists in the United States identified factors associated with guideline-consistent antibiotic prescribing to be postgraduate education, urban locale, and a smaller patient panel.^60^ Reasons for higher antibiotic prescribing rates included increasing use of dental implants, an aging population, underinsurance driving antibiotics as an oral surgery substitute, slow adoption of new guidelines, lack of awareness of the role of dentists in antibiotic resistance, and physician and patient pressure.^50,61^ These characteristics are similar to those associated with physician antibiotic overprescribing.^62^ Therefore, antibiotic stewardship strategies shown to be effective in outpatient medical clinics may also improve antibiotic prescribing in dentistry. In fact, a recent example has provided early evidence that implementing the Centers for Disease Control and Prevention’s Core Elements of Outpatient Antibiotic Stewardship in dental practices was effective.^63^

### Limitations and Public Health Implications

These results are not without limitations. The cohort is a convenience sample of US patients with commercial dental insurance. Therefore, our results may not be representative of the uninsured and underrepresents persons with Medicaid and Medicare benefits. Medicare does not generally cover dental care, and the state provision of dental benefits to adults with Medicaid is optional.^64,65^ As a result, these persons are not included in our sample unless supplemental commercial dental benefits were purchased. Due to limitations in the data set, the prescriptions cannot be directly linked with the health care encounter or prescriber. We adapted methods used by other investigators to link antibiotic prescriptions to health care encounters and exclude other indications for antibiotics. To increase the specificity of our antibiotic-related dental visits, we conservatively defined a cohort in which other indications for antibiotics are unlikely. In contrast to medical care providers, dentists rarely use diagnostic codes (*ICD-9* or *ICD-10-CM*) and are reimbursed based on procedure codes (*CDT*).^66,67^ Therefore, it is difficult to associate a diagnosis with the prescription. To increase the likelihood that an antibiotic was prescribed for infection prophylaxis (vs treatment of an oral infection), the days’ supply for the antibiotic prescription was limited to 2 days or less (whereas oral infections are likely to be treated for ≥3 days). We did not assess dental visits at which an antibiotic was indicated but not dispensed. Although the 2007 infective endocarditis guidelines significantly decreased the number of patients with a prophylaxis indication,^5^ data are conflicting regarding the association of this change with the incidence of endocarditis.^28,29,68,69,70^ However, the sole study^70^ with results identifying an increase in infective endocarditis in the United States did not include dental visit data and was not able to adjust for increases in infective endocarditis observed with the opioid epidemic. So, it is difficult to elucidate the causal factor (dental visit vs substance misuse). We did not apply 2017 (after our study period) expert panel recommendations of clinical scenarios in which antibiotic prophylaxis may be appropriate in patients with prosthetic joints.^71^ All scenarios include patients with multiple comorbidities (eg, prosthetic joint plus uncontrolled diabetes and prosthetic joint plus immunocompromised state). Because the comorbidities in the expert panel recommendations^71^ are similar to those in the 2003 prosthetic joint guidelines,^25^ we anticipate the results from the expert panel recommendations to be similar to the results from the 2003 prosthetic joint guidelines. Due to the difficulty in identifying specific diagnoses, we broadened the definition for certain conditions to include all cardiac transplantation (removing cardiac valvulopathy), all diabetes (vs just type 1), and all congenital heart diseases. Therefore, unnecessary prescribing is likely higher than our results indicate.

These findings have strong public health implications. To our knowledge, this is the first national analysis of overprescribing of antibiotics for infection prophylaxis before dental procedures and should initiate a call to action to the public health and dental communities to improve prescribing of antibiotics for infection prophylaxis. Because dentists primarily prescribe antibiotics for infection prophylaxis,^49^ a decrease in unnecessary antibiotic prophylaxis will significantly decrease overall antibiotic prescribing by dentists. Therefore, specific antibiotic stewardship strategies and prescribing tools targeted to dentists and dental practices should be developed, implemented, and assessed for effectiveness in improving prescribing of antibiotics for infection prophylaxis before dental procedures.

## Conclusions

More than 80% of antibiotic prophylaxis prescriptions before dental procedures are unnecessary: clindamycin use, the presence of prosthetic joints, and residence in the western United States were associated with unnecessary prescribing. While antibiotic prophylaxis is appropriately prescribed for indicated dental procedures in patients with cardiac conditions, most antibiotic prophylaxis is prescribed to patients in whom guideline-identified risk factors are not present. Although prescribing is slowly improving, the high proportion of antibiotics that were found to be unnecessary in our study is worrisome. Implementing antimicrobial stewardship efforts in dental practices is an opportunity to improve antibiotic prescribing for infection prophylaxis.

## Notes

Supplement. **eTable 1.** Multivariable Analysis on Factors Associated With Unnecessary Antibiotic Prophylaxis Based on the 2003 AAOS/ADA Guidelines (n = 168 420) **eTable 2.** Multivariable Analysis on Factors Associated With Unnecessary Antibiotic Prophylaxis Excluding Patients With a Prosthetic Joint (n = 96 769) **eTable 3.** Multivariable Analysis on Factors Associated With Unnecessary Antibiotic Prophylaxis in Patients With a Prosthetic Joint (n = 71 651)
