# Supplementary material for: Assessment of the Appropriateness of Antibiotic Prescriptions for Infection Prophylaxis Before Dental Procedures, 2011 to 2015
Source: JAMA Netw Open. 2019 May 31;2(5):e193909. doi: 10.1001/jamanetworkopen.2019.3909 (PMC6547109; doi:10.1001/jamanetworkopen.2019.3909)
Supplement: Supplement. — eTable 1. Multivariable Analysis on Factors Associated With Unnecessary Antibiotic Prophylaxis Based on the 2003 AAOS/ADA Guidelines (n = 168 420) eTable 2. Multivariable Analysis on Factors Associated With Unnecessary Antibiotic Prophylaxis Excluding Patients With a Prosthetic Joint (n = 96 769) eTable 3. Multivariable Analysis on Factors Associated With Unnecessary Antibiotic Prophylaxis in Patients With a Prosthetic Joint (n = 71 651) [file jamanetwopen-2-e193909-s001.pdf]

Supplementary Online Content

Suda KJ, Calip GS, Zhou J, et al. Assessment of the appropriateness of antibiotic prescriptions for infection prophylaxis before dental procedures, 2011 to 2015. *JAMA Netw Open*. 2019;2(5):e193909. doi:10.1001/jamanetworkopen.2019.3909

eTable 1. Multivariable Analysis on Factors Associated With Unnecessary Antibiotic Prophylaxis Based on the 2003 AAOS/ADA Guidelines (n=168,420)

eTable 2. Multivariable Analysis on Factors Associated With Unnecessary Antibiotic Prophylaxis Excluding Patients With a Prosthetic Joint (n=96,769)

eTable 3. Multivariable Analysis on Factors Associated With Unnecessary Antibiotic Prophylaxis in Patients With a Prosthetic Joint (n=71,651)

This supplementary material has been provided by the authors to give readers additional information about their work.

eTable 1. Multivariable Analysis on Factors Associated With Unnecessary Antibiotic Prophylaxis Based on the 2003 AAOS/ADA Guidelines (n=168,420)

Characteristic	OR (95% CI)
**Year effect**	
**2014-2015 vs. 2011-2013** *(reference)*	0.89 (0.87-0.91)
**Age** at visit, years	
18-34	1 [Reference]
35-44	0.90 (0.82-0.99)
45-54	0.92 (0.85-1.00)
55-64	0.83 (0.77-0.90)
65+	0.67 (0.62-0.73)
**Female sex** *(reference=male)*	1.19 (1.15-1.23)
**Region**	
Northeast	0.81 (0.78-0.85)
Midwest	1 [Reference]
South	0.88 (0.85-0.91)
West	1.16 (1.08-1.24)
Unknown	1.80 (0.68-4.77)
**ADA dental procedure category**[a]	
Diagnostic	0.90 (0.88-0.92)
Preventive	0.63 (0.61-0.65)
Restorative	0.99 (0.96-1.02)
Oral and maxillofacial surgery	0.73 (0.70-0.76)
Periodontics	0.65 (0.62-0.68)
Adjunctive general services	1.46 (1.35-1.59)
Endodontics	0.61 (0.58-0.65)
Implant services	1.80 (1.58-2.05)
Prosthodontics	1.70 (1.49-1.94)
Orthodontics	3.92 (1.16-13.25)
Maxillofacial prosthetics	1.71 (1.12-2.63)
**Antibiotic prescribed**	
Amoxicillin	1 [Reference]
Clindamycin	1.07 (1.03-1.12)
Other[b]	1.53 (1.47-1.60)
Rural vs. Urban *(reference)*	
Rural	0.86 (0.82-0.90)
**Pre-index health service utilization**[c]	
PCP visits (Yes/No)	0.96 (0.94-0.98)
Specialist visits (yes/No)	0.84 (0.82-0.86)
ER visits (yes/No)	0.91 (0.88-0.93)
Admission (yes/No)	0.80 (0.78-0.82)

[a]The American Dental Association (ADA) has a standardized system to group Comprehensive Dental Terminology (CDT) codes (dental procedure codes) into categories (shown in the table). There could be multiple procedures performed during the same visit. The ADA does not include CPT and Healthcare Common Procedure Coding System (HCPCS) codes in their standard ADA dental procedure categories. CPT and HCPCS codes are included in 'Category not available'.

[c]Other antibiotics include: Ampicillin, Cefaclor, Cefadroxil, Cefazolin, Cefdinir, Cefixime, Cefoxitin, Cefpodoxime, Cefprozil, Ceftazidime, Ceftriaxone, Cefuroxime, Ciprofloxacin, Clarithromycin, Demeclocycline, Dicloxacillin, Erythromycin, Gemifloxacin, Levofloxacin, Lincomycin, Linezolid, Minocycline, Moxifloxacin, Ofloxacin, Sulfamethoxazole/trimethoprim, Tetracycline, Trimethoprim, Vancomycin

[c]Number of health service utilization assessed over the 6-month pre-dental visit period, not accounting for enrollment in dental or medical plans. We defined outpatient clinic visits with a provider type of nurse practitioners, physician assistant, or medical doctors. Medical doctors with a specialty of internal medicine or family medicine were included as primary care providers (PCP). Other types of clinical encounters were defined as a specialist visit and may include health care encounters without a medical provider (e.g., nurse visit or laboratory visit). ER=visits to the emergency room/department.

© 2019 Suda KJ et al. *JAMA Network Open*.

eTable 2. Multivariable Analysis on Factors Associated With Unnecessary Antibiotic Prophylaxis Excluding Patients With a Prosthetic Joint (n=96,769)

Characteristic	OR (95% CI)
**Age** at visit, years	
18-34 (reference)	1 [Reference]
35-44	0.86 (0.76-0.97)
45-54	0.81 (0.72-0.90)
55-64	0.79 (0.71-0.88)
65+	0.63 (0.57-0.71)
**Female sex** *(reference=male)*	1.30 (1.24-1.36)
**Region**	
Northeast	0.80 (0.74-0.85)
Midwest	1 [Reference]
South	0.91 (0.86-0.96)
West	1.19 (1.06-1.35)
Unknown	1.89 (0.61-5.93)
**Year of visit**	
2011	1 [Reference]
2012	0.84 (0.81-0.87)
2013	0.72 (0.70-0.75)
2014	0.62 (0.59-0.65)
2015	0.56 (0.53-0.58)
**ADA dental procedure category**[a]	
Diagnostic	0.91 (0.88-0.94)
Preventive	0.58 (0.56-0.61)
Restorative	0.96 (0.91-1.01)
Oral and maxillofacial surgery	0.69 (0.65-0.74)
Periodontics	0.63 (0.59-0.67)
Adjunctive general services	1.60 (1.39-1.84)
Endodontics	0.62 (0.58-0.66)
Implant services	1.54 (1.31-1.82)
Prosthodontics	1.72 (1.39-2.13)
Orthodontics	2.83 (0.82-9.81)
Maxillofacial prosthetics	1.24 (0.92-1.67)
**Antibiotic prescribed**	
Amoxicillin	1 [Reference]
Clindamycin	1.15 (1.07-1.23)
Other[b]	1.94 (1.80-2.09)
Rural vs. Urban *(reference)*	
Rural	0.87 (0.82-0.93)
**Pre-index health service utilization**[c]	
PCP visits (Yes/No)	0.95 (0.93-0.97)
Specialist visits (yes/No)	0.86 (0.83-0.88)
ER visits (yes/No)	0.95 (0.92-0.98)
Admission (yes/No)	0.80 (0.76-0.84)

[a]The American Dental Association (ADA) has a standardized system to group Comprehensive Dental Terminology (CDT) codes (dental procedure codes) into categories (shown in the table). There could be multiple procedures performed during the same visit. The ADA does not include CPT and Healthcare Common Procedure Coding System (HCPCS) codes in their standard ADA dental procedure categories. CPT and HCPCS codes are included in 'Category not available'.

[b]Other antibiotics include: Ampicillin, Cefaclor, Cefadroxil, Cefazolin, Cefdinir, Cefixime, Cefoxitin, Cefpodoxime, Cefprozil, Ceftazidime, Ceftriaxone, Cefuroxime, Ciprofloxacin, Clarithromycin, Demeclocycline, Dicloxacillin, Erythromycin, Gemifloxacin, Levofloxacin, Lincomycin, Linezolid, Minocycline, Moxifloxacin, Ofloxacin, Sulfamethoxazole/trimethoprim, Tetracycline, Trimethoprim, Vancomycin

[c]Number of health service utilization assessed over the 6-month pre-dental visit period, not accounting for enrollment in dental or medical plans. We defined outpatient clinic visits with a provider type of nurse practitioners, physician assistant, or medical doctors. Medical doctors with a specialty of internal medicine or family medicine were included as primary care providers (PCP). Other types of clinical encounters were defined as a specialist visit and may include health care encounters without a medical provider (e.g., nurse visit or laboratory visit). ER=visits to the emergency room/department.

© 2019 Suda KJ et al. *JAMA Network Open*.

eTable 3. Multivariable Analysis on Factors Associated With Unnecessary Antibiotic Prophylaxis in Patients With a Prosthetic Joint (n=71,651)

**Characteristic**[a]	**OR (95% CI)**
**Age** at visit, years	
18-34 (reference)	1 [Reference]
35-44	1.58 (0.97-2.57)
45-54	1.54 (1.00-2.38)
55-64	1.22 (0.80-1.86)
65+	0.82 (0.53-1.25)
**Female sex** *(reference=male)*	1.03 (0.96-1.09)
**Year of visit**	
2011 (reference)	1 [Reference]
2012	0.79 (0.74-0.84)
2013	0.67 (0.63-0.72)
2014	0.56 (0.52-0.61)
2015	0.52 (0.48-0.56)
**Antibiotic prescribed**	
Amoxicillin (reference)	1 [Reference]
Clindamycin	1.0 (0.93-1.07)
Other[b]	1.14 (1.05-1.23)
Rural vs. Urban *(reference)*	
Rural	0.65 (0.61-0.70)
**Pre-index health service utilization**[c]	
PCP visits (Yes/No)	0.99 (0.95-1.02)
Specialist visits (yes/No)	0.94 (0.90-0.98)
ER visits (yes/No)	0.86 (0.82-0.90)
Admission (yes/No)	0.94 (0.90-0.98)

[a]Due to the small sample size, the model converged after removing variables with sparse data.
[b]Other antibiotics include: Ampicillin, Cefaclor, Cefadroxil, Cefazolin, Cefdinir, Cefixime, Cefoxitin, Cefpodoxime, Cefprozil, Ceftazidime, Ceftriaxone, Cefuroxime, Ciprofloxacin, Clarithromycin, Demeclocycline, Dicloxacillin, Erythromycin, Gemifloxacin, Levofloxacin, Lincomycin, Linezolid, Minocycline, Moxifloxacin, Ofloxacin, Sulfamethoxazole/trimethoprim, Tetracycline, Trimethoprim, Vancomycin
[c]Number of health service utilization assessed over the 6-month pre-dental visit period, not accounting for enrollment in dental or medical plans. We defined outpatient clinic visits with a provider type of nurse practitioners, physician assistant, or medical doctors. Medical doctors with a specialty of internal medicine or family medicine were included as primary care providers (PCP). Other types of clinical encounters were defined as a specialist visit and may include health care encounters without a medical provider (e.g., nurse visit or laboratory visit). ER=visits to the emergency room/department.

© 2019 Suda KJ et al. *JAMA Network Open*.
